# Soil Bacterial and Fungal Communities Exhibit Distinct Long-Term Responses to Disturbance in Temperate Forests

**DOI:** 10.3389/fmicb.2019.02872

**Published:** 2019-12-11

**Authors:** Ernest D. Osburn, Steven G. McBride, Frank O. Aylward, Brian D. Badgley, Brian D. Strahm, Jennifer D. Knoepp, J. E. Barrett

**Affiliations:** ^1^Department of Biological Sciences, College of Science, Virginia Polytechnic Institute and State University, Blacksburg, VA, United States; ^2^School of Plant and Environmental Sciences, College of Agriculture and Life Sciences, Virginia Polytechnic Institute and State University, Blacksburg, VA, United States; ^3^Department of Forest Resources and Environmental Conservation, College of Natural Resources and Environment, Virginia Polytechnic Institute and State University, Blacksburg, VA, United States; ^4^Coweeta Hydrologic Laboratory, USDA Forest Service Southern Research Station, Otto, NC, United States

**Keywords:** soil, microbial community, forest management, qPCR, 16S, ITS

## Abstract

In Appalachian ecosystems, forest disturbance has long-term effects on microbially driven biogeochemical processes such as nitrogen (N) cycling. However, little is known regarding long-term responses of forest soil microbial communities to disturbance in the region. We used 16S and ITS sequencing to characterize soil bacterial (16S) and fungal (ITS) communities across forested watersheds with a range of past disturbance regimes and adjacent reference forests at the Coweeta Hydrologic Laboratory in the Appalachian mountains of North Carolina. Bacterial communities in previously disturbed forests exhibited consistent responses, including increased alpha diversity and increased abundance of copiotrophic (e.g., Proteobacteria) and N-cycling (e.g., Nitrospirae) bacterial phyla. Fungal community composition also showed disturbance effects, particularly in mycorrhizal taxa. However, disturbance did not affect fungal alpha diversity, and disturbance effects were not consistent at the fungal class level. Co-occurrence networks constructed for bacteria and fungi showed that disturbed communities were characterized by more connected and tightly clustered network topologies, indicating that disturbance alters not only community composition but also potential ecological interactions among taxa. Although bacteria and fungi displayed different long-term responses to forest disturbance, our results demonstrate clear responses of important bacterial and fungal functional groups (e.g., nitrifying bacteria and mycorrhizal fungi), and suggest that both microbial groups play key roles in the long-term alterations to biogeochemical processes observed following forest disturbance in the region.

## Introduction

Globally, land use change has modified 75% of ice-free terrestrial ecosystems (Ellis, 2011), with conversion of forests to managed states (e.g., agriculture, timber plantations) being one of earth’s dominant land conversions (Vitousek et al., 1997; Foley et al., 2005; Rudel et al., 2005). Although ∼50% of Earth’s land surface was forested in prehistoric times, approximately 40% of that forest cover has been lost and much of the remaining forest subjected to various forms of disturbance, especially within the past two centuries (Millenium Ecosystem Assessment, 2005). The extent of anthropogenic forest conversion continues to accelerate in the 21st Century (Drummond and Loveland, 2010; Hansen et al., 2010; Watson et al., 2014), highlighting the need to characterize impacts of forest disturbance on terrestrial biodiversity and ecosystem functions. Understanding these impacts is particularly critical from a biogeochemical perspective, as forested ecosystems are central components of Earth’s elemental cycles and provide ecosystem services that support human well-being, including storage of carbon (C), regulation of nutrient cycles, and provisioning of clean drinking water (Millenium Ecosystem Assessment, 2005). These biogeochemical processes and associated ecosystem services are primarily driven by soil microorganisms, which perform a variety of essential functions including litter decomposition and several C- and nitrogen (N)-cycling processes (Fierer, 2017), thus emphasizing the need for studies investigating effects of forest disturbance on soil microbial communities.

Forest disturbances influence multiple factors that can affect terrestrial microorganisms, including vegetation characteristics (i.e., plant biomass and species composition) as well as several soil physicochemical properties. For example, disturbance alters forest soil C and N stocks (Guo and Gifford, 2002; Foote et al., 2015; James and Harrison, 2016), both of which are known drivers of microbial community structure (e.g., Eilers et al., 2010; Ramirez et al., 2012). Several previous studies have directly assessed effects of forest disturbances on soil microbial communities: timber harvest (Kohout et al., 2018; Mushinski et al., 2018a, b), conversion to agriculture (Jangid et al., 2011; Rodrigues et al., 2013; Zhou et al., 2018), and prescribed fire (Oliver et al., 2015; Shen et al., 2016) can all alter bacterial and/or fungal communities. Additionally, a recent global meta-analysis showed consistent bacterial community changes in previously disturbed forests, including increased relative abundance of r-selected bacterial phyla (e.g., Proteobacteria) with past disturbance (Zhou et al., 2018). Although these prior studies suggest that forest soil microbial community shifts in response to disturbance are likely to occur, no studies to our knowledge have fully characterized long-term (i.e., several decades) responses of both soil bacterial and fungal communities to several different past disturbances (e.g., timber harvest, agricultural conversion, and timber plantation conversion) simultaneously in temperate forests.

Understanding disturbance responses of soil microbial communities is particularly important in forests of the Appalachian region of the Eastern United States, where approximately 70% of land area is forested (Simon et al., 2005) and nearly all forested ecosystems in the region have experienced past disturbances from human activities, including commercial logging and/or conversion to agriculture (Gragson and Bolstad, 2006). Further, forest disturbance is known to have long-term effects on biogeochemical cycling in these ecosystems; previous studies from the region have shown impacts of disturbance on watershed-scale N-cycling (Vitousek et al., 1979; Peterjohn et al., 1996; Swank and Vose, 1997), with previously disturbed forested watersheds often exporting N at elevated rates for several decades following disturbance (Webster et al., 2016). Some prior studies in the region assessed long-term effects of disturbance on soil microbial N-cycle functions, finding elevated nitrification rates (Montagnini et al., 1986; Keiser et al., 2016) and elevated abundance of nitrifying microorganisms (Lin et al., 2017) in previously disturbed forests. However, though one previous study documented effects of previous logging and conversion to agriculture on PLFA-determined soil microbial community structure in the Appalachians (Fraterrigo et al., 2006), relatively little is known regarding long-term disturbance impacts on bacterial and fungal communities in the region.

To address questions of long-term soil microbial responses to past forest disturbance, we characterized soil bacterial and fungal communities from four previously disturbed forested watersheds as well as adjacent reference forests at the Coweeta Hydrologic Laboratory in the Appalachian mountains of North Carolina. Because bacterial community structure is driven primarily by soil physicochemical variables (Fierer and Jackson, 2006) and because these variables (e.g., soil ${\text{NO}}_{3}^{-}$) respond similarly to different disturbances in the region (e.g., Keiser et al., 2016), we predicted that bacterial communities would display generally consistent responses across different past disturbances. More specifically, we predicted higher relative abundance of bacterial taxa associated with N-cycling processes in previously disturbed forests (i.e., nitrifiers) and that elevated soil inorganic-N in disturbed forests would promote higher relative abundance of r-selected (i.e., copiotrophic) soil bacterial taxa. In contrast, because fungal communities are often closely coupled to plant communities (Bonfante and Anca, 2009; Peay et al., 2013), and because different forest conversions have unique effects on plant communities, we predicted fungal responses to be site-specific. For example, we predicted pine conversion to increase abundance of ectomycorrhizal (ECM) fungi, while disturbances that promote arbuscular mycorrhizal (AM) hosts (e.g., red maple and tulip poplar) to increase abundance of AM fungi.

## Materials and Methods

### Site Description and Soil Sampling

**FIGURE 1:**
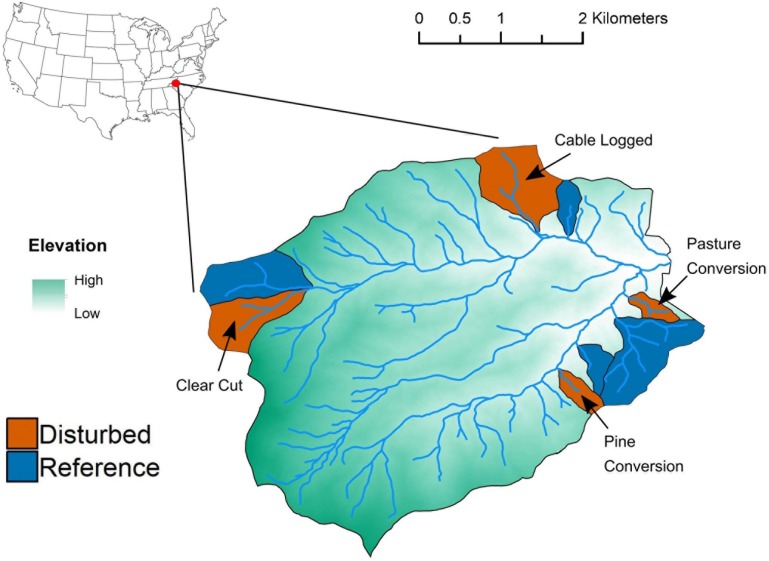
Map of disturbed-reference watershed pairs at the USDA Forest Service Coweeta Hydrologic Laboratory sampled for this study.

We conducted this study at the Coweeta Hydrologic Laboratory, a USDA Forest Service experimental forest located in the Blue Ridge physiographic province in the Appalachian Mountains of southwestern North Carolina (latitude 35°03′ N, longitude 83°25′ W). Within the Coweeta Basin, we selected four forested watersheds that experienced whole-watershed disturbances associated with forest management experiments conducted by the USDA Forest Service at different times during the 20th Century. All disturbed watersheds are currently forested and have not been manipulated since disturbances occurred approximately four to eight decades ago (Table 1). Previous disturbances included clear-cutting, commercial clear cut-cable logging, conversion to pasture, and conversion to pine monoculture (Table 1 and Figure 1). Adjacent to each previously disturbed watershed is a reference watershed (Figure 1) that has not been disturbed since the time period 1919–1923, when ∼20% of the basal area of the entire Coweeta Basin was cut and harvested (Elliott and Vose, 2011). Detailed information, including size, aspect, and dominant vegetation of each watershed can be found in Supplementary Table S1. Within each of the eight watersheds, we established six 4 m × 4 m plots (48 plots total) at 40 m intervals along a 200-m stretch of the main stream channel. All plots were located 5 m upslope from the stream itself. We sampled near-stream environments because these areas support high rates of microbial biogeochemical processes (Knoepp and Clinton, 2009) and because spatially consistent sampling across watersheds of varying sizes (Supplementary Table S1) enabled cross-watershed comparisons. In June 2018, at the height of the growing season, we surveyed all woody vegetation and sampled five soil cores (from four plot corners and plot center) from each plot. Soil samples included the top 10 cm of mineral soil and did not include O-horizon material. This sampling depth generally includes the entire A horizon in low elevation watersheds (Knoepp and Swank, 1994) and coincides with the depth of sampling in many research studies in the Coweeta Basin (e.g., Knoepp et al., 2018; Osburn et al., 2018). We composited samples by plot, sieved composited samples (4 mm), and stored subsamples at −20°C (for DNA extraction) or 4°C (for measurement of soil properties) until further processing.

**TABLE 1:** Names and disturbance histories of watersheds sampled in this study.

**Name**	**Disturbance history**
Cable logged	Commercially clear-cut and cable-logged in 1977
Clear cut	All woody vegetation cut in 1963, no products removed
Pasture conversion	Clear-cut and planted to grass in 1958, limed and fertilized in 1959, fertilized in 1965, grass herbicided 1966–1967
Pine conversion	All woody vegetation cut in 1940, re-growth cut annually until 1955, white pine planted in 1956
All references	Undisturbed since at least 1923

### Soil Properties

Soil pH was measured in a 1:1 soil:DI H_2_O mixture using a Hach Sension+ pH meter (Hach Company, Loveland, CO, United States). Soils were extracted for 1 h with 2 M KCl (1:5 soil:solution ratio) and extracts were analyzed for ${\text{NH}}_{4}^{+}$ and ${\text{NO}}_{3}^{-}$ using a Lachat QuikChem flow injection analyzer (Hach Company, Loveland, CO, United States). Microbial Biomass C and N were determined using a modified chloroform extraction method (Fierer and Schimel, 2003) and extracts were measured for dissolved organic carbon (DOC), total dissolved N, and microbial biomass C and N on an Elementar vario cube TOC/TN (Elementar Americas Inc., Mt. Laurel, NJ, United States). Soil subsamples were air dried, milled, and analyzed for total C and total N using an Elementar vario MAX cube (Elementar Americas Inc., Mt. Laurel, NJ, United States). Microbial activity was assessed via substrate-induced respiration (SIR) (Bradford et al., 2008). Gravimetric water content was measured by mass loss after oven drying at 105°C for 24 h and all soil properties are presented on an oven-dried basis.

### DNA Extraction and qPCR

DNA was extracted from ∼0.25 g fresh soil using the Qiagen DNeasy PowerSoil kit (Qiagen, Valencia, CA, United States) and extracts were quantified using a Qubit 2.0 Fluorometer (Thermo Fisher Inc., Waltham, MA, United States). We estimated bacterial and fungal abundance by qPCR amplifying the 16S rRNA and ITS regions, respectively. For 16S rRNA qPCR we used the EUB 338/515 primer set while for ITS qPCR we used the ITS1f/5.8s primers (Fierer et al., 2005). Each qPCR reaction contained 10 μl Quantitect SYBR green master mix (Qiagen, Valencia, CA, United States), 2 μl of 1:10 diluted DNA template (∼1–4 ng DNA), 0.25 μM forward and reverse primers, and nuclease-free H_2_O to 20 μl. For both genes, thermal cycling conditions were as follows: 15 min at 95°C followed by 40 cycles of 15 s at 94°C, 30 s at 55°C, and 30 s at 72°C. Standard curves were generated by amplifying serial dilutions of the target regions cloned into plasmids, with amplification efficiencies ranging from 87 to 92% and *R*^2^ values >0.99. All amplifications were performed in triplicate and amplification specificity was assessed using melt curve analysis. 16S and ITS gene copy numbers were corrected for dry soil mass and fungal:bacterial ratios (ITS:16S) were calculated by dividing fungal gene copy numbers by bacterial gene copy numbers (Fierer et al., 2005).

### 16S and ITS Sequencing and Bioinformatic Analysis

We characterized bacterial and fungal communities by amplifying and sequencing the V4 16S rRNA region and the ITS1 region, respectively. We amplified the V4 region using the 515F/806R primer pair (Apprill et al., 2015; Parada et al., 2016) and the ITS region using the ITS1F/ITS2 primer pair (Bellemain et al., 2010). We amplified samples in triplicate and PCR reactions contained 10 μl Thermo Fisher Platinum II Hot Start PCR Master Mix (Thermo Fisher Inc., Waltham, MA, United States), 1 μl undiluted DNA template (∼5–20 ng DNA), 0.2 μM forward and reverse primer, and nuclease-free H_2_O to 25 μl. We also amplified negative controls for each barcoded PCR primer to detect possible contamination. Thermal cycling conditions for 16S amplification were 2 min at 94°C followed by 35 cycles of 45 s at 94°C, 60 s at 50°C, and 90 s at 72°C, with a 10 min final extension at 72°C. Conditions for ITS amplification were 2 min at 94°C followed by 35 cycles of 30 s at 94°C, 30 s at 52°C, and 30 s at 68°C, with a 10 min final extension at 68°C. After amplification, we pooled triplicate PCR amplicons, visualized amplicons and negative controls on an agarose gel, and purified them using the Qiagen QIAquick PCR Purification Kit (Qiagen, Valencia, CA, United States). We quantified purified amplicons (see above) and pooled 16S and ITS amplicons separately in equimolar ratios. Amplicons were sequenced on the Illumina MiSeq platform using 250 bp paired-end reads. Due to poor quality scores for the ITS forward reads, we only processed and analyzed the reverse reads. Raw sequence reads were deposited in NCBI’s BioProject database under accession number PRJNA548911.

We processed raw sequence reads using the QIIME2 pipeline (Bolyen et al., 2018). After demultiplexing, we joined paired-ends (16S only), denoised sequences, and removed chimeras using DADA2 (Callahan et al., 2016). We then used VSEARCH (Rognes et al., 2016) to cluster processed sequences into 97% OTUs and removed OTUs only appearing in one sample. After processing, we retained 2,511,186 sequences and 794,909 sequences for 16S and ITS, respectively. For downstream statistical analyses, we randomly selected 12,754 16S sequences and 7,038 ITS sequences from each sample to account for differences in sequencing depth. One sample was excluded from ITS sequence analysis due to insufficient sequencing depth. After random sampling, we retained 2,560 16S OTUs and 1,419 ITS OTUs for further analysis.

We assigned taxonomy to sequences using a naïve-Bayes classifier (Pedregosa et al., 2011) trained on the Greengenes and UNITE databases for 16S and ITS, respectively (Abarenkov et al., 2010; McDonald et al., 2012). To assess potential bacterial life history shifts induced by disturbance, we categorized bacterial phyla as copiotrophic (i.e., r-selected, Proteobacteria + Bacteroidetes) or oligotrophic (i.e., K-selected, Acidobacteria + Actinobacteria) similar to the approach used by Zhou et al. (2018), and calculated copiotroph:oligotroph ratios for each sample. For functional analysis of fungal communities, we parsed fungal OTUs into functional guilds using FUNGuild (Nguyen et al., 2016). Similar to previous studies (e.g., Veach et al., 2017), we only analyzed sequences assigned to a single guild with a confidence of “probable” or “highly probable.”

### Statistical Analysis

All statistical analyses were performed in R (R Core Development Team, 2017) using the “phyloseq,” “vegan,” “emmeans,” and “MASS” packages (McMurdie and Holmes, 2013; Lenth et al., 2019; Oksanen et al., 2019; Ripley et al., 2019). For all statistical analyses, *P* < 0.05 was considered statistically significant while *P* < 0.1 was considered marginally significant, and all plots were considered independent replicates. We determined 16S and ITS alpha (i.e., Shannon) diversity using the “estimate_richness” function in the phyloseq package and identified differentially abundant OTUs across disturbed and reference forests using the “exactTest” function in the edgeR package (Robinson et al., 2010). To determine disturbance effects on Shannon diversity, copiotroph:oligotroph ratios, and relative abundance of phyla/classes/guilds, we used two-way ANOVAs with disturbance and watershed pair as factors in the models. Although we focus on main effects of disturbance from the ANOVAs, using watershed pair as a factor allowed us to determine pairwise differences between watersheds within each disturbed-reference pair using the emmeans package (“emmeans” and “contrast” functions), though these pairwise differences should be interpreted with caution, as individual disturbances were not replicated. Where necessary, we log-transformed variables in order to meet assumptions of normality of residuals. When log transformation failed to normalize residuals, we verified ANOVA results using generalized linear models (“glm” function with gamma distribution and log-link function, MASS package).

We visualized 16S and ITS community structure using Non-Metric Multidimensional Scaling (NMDS, “metaMDS” function, vegan package) with Bray–Curtis distance matrices (untransformed) and added key soil variables to the ordinations using the “envfit” function (vegan package). We determined effects of disturbance on 16S and ITS community structure using PERMANOVA (“adonis2” function, vegan package). We also used PERMANOVA to determine pairwise differences between watersheds in each disturbed-reference pair, with *P*-values adjusted using the Benjamini–Hochberg method to control for false discovery rate (Benjamini and Hochberg, 1995). We used variation partitioning (Peres-Neto et al., 2006) to determine relationships between microbial communities, soil properties, and vegetation communities, and used distance-based redundancy analysis (“dbrda” function, Bray–Curtis distances, vegan package) to test the statistical significance of each partition.

To investigate responses of potential microbial community interactions to disturbance, we constructed bacterial and fungal co-occurrence networks for reference and disturbed forests separately by grouping communities from all 24 samples from each treatment. Similar to Shi et al. (2016), to ensure robustness of correlations used to construct networks, we only included OTUs that occurred in a minimum of 10 samples for each treatment. Spearman’s rank correlations were used to calculate interaction strength among OTUs and network metrics were calculated using all significant OTU correlations (*P* < 0.01 and |ρ| > 0.5). For visualization purposes, we constructed random networks using code modified from Williams et al. (2014) available at https://github.com/ryanjw/co-occurrence. We then used the igraph package (Csardi and Nepusz 2006) to calculate degree centrality, closeness centrality, and betweenness centrality, all of which were normalized for each respective network. We also used igraph to calculate clustering coefficients for each node in each network. We identified differences in network topology (degree centrality, closeness centrality, betweenness centrality, and clustering coefficients) between disturbed and reference communities using Kruskal–Wallis tests, similar to the approach taken by Ma et al. (2016), while proportion of negative edges was compared using *Z*-tests. Although statistical comparisons were performed on the full networks including all significant correlations, for purposes of visualization, our bacterial network diagrams only include correlations with |ρ| > 0.7.

## Results

### Soil Properties and Plant Communities

Disturbed forest soils were characterized by ∼41% higher ${\text{NH}}_{4}^{+}$ concentrations, ∼12% higher SIR, significantly higher pH, and ∼900% higher ${\text{NO}}_{3}^{-}$ concentration relative to reference forest soils (all ANOVA *P* < 0.05, Supplementary Table S2). In contrast, reference soils had ∼29% higher DOC concentrations, ∼18% higher microbial biomass C, ∼11% higher C:N ratios, and ∼19% higher DOC:TDN ratios (all ANOVA *P* < 0.05, Supplementary Table S2) relative to disturbed soils. Vegetation surveys revealed distinct plant communities between reference and disturbed forests and also showed a significant disturbance × watershed pair interaction (both PERMANOVA *P* < 0.001, Supplementary Figure S1), indicating unique effects of particular disturbance history on forest vegetation communities. In general, our vegetation analysis reflects known disturbance effects on woody vegetation previously described from Coweeta (i.e., increased abundance of species such as red maple and tulip poplar, see Supplementary Table S1) (Elliott and Vose, 2011).

### Bacterial and Fungal Abundance, Copiotroph:Oligotroph Ratios, and α Diversity

**FIGURE 2:**
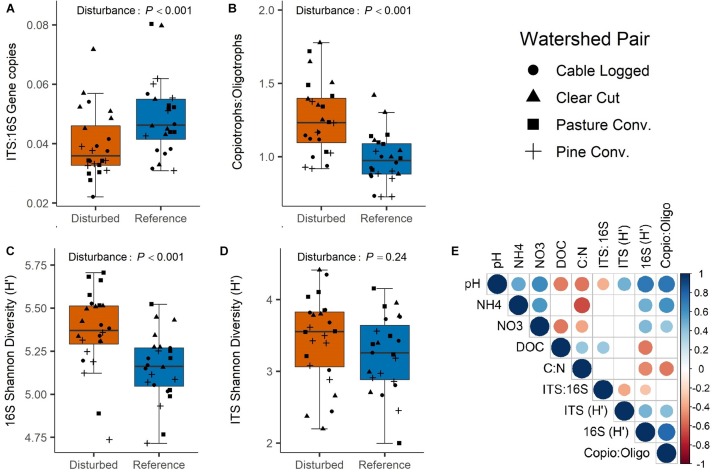
Effects of disturbance on ITS:16S gene copies **(A)**, bacterial Copiotroph:Oligotroph ratios **(B)**, 16S Shannon Diversity **(C)**, and ITS Shannon Diversity **(D)**. *P*-values are overall disturbance effects from two-way ANOVA. Correlogram **(E)** visualizes Spearman rank correlation coefficients between microbial variables from panels **A** to **D** and key soil variables (only statistically significant correlations shown, *P* < 0.05).

Bacterial (16S) gene copy abundance was marginally higher in disturbed watersheds (ANOVA *P* = 0.07, Supplementary Figure S2A), while fungal (ITS) gene copy abundance was not affected by disturbance (ANOVA *P* = 0.54, Supplementary Figure S2B). ITS:16S gene copy ratios were ∼23% higher in reference than in disturbed soils (ANOVA *P* < 0.001, Figure 2A), which was driven primarily by large differences between reference and disturbed forests in the pasture conversion and pine conversion watershed pairs (Supplementary Figure S2C). ITS:16S gene copy ratios were negatively correlated with soil pH and positively correlated with soil DOC (Figure 2E). Disturbed soils had ∼29% higher bacterial copiotroph:oligotroph ratios than reference soils (ANOVA *P* < 0.001, Figure 2B), a pattern that was consistent across all four disturbed-reference watershed pairs (Supplementary Figure S3). Copiotroph:oligotroph ratios were positively correlated with soil pH, ${\text{NO}}_{3}^{-}$, and ${\text{NH}}_{4}^{+}$, and negatively correlated with soil C:N ratios (Figure 2E).

Bacterial Shannon diversity was significantly higher in disturbed forest soils (ANOVA *P* < 0.001, Figure 2C), which was generally consistent across all four past disturbances, but was most prominent in the pasture conversion disturbed-reference pair (Supplementary Figure S4A). Bacterial Shannon diversity was positively correlated with soil pH, ${\text{NO}}_{3}^{-}$, and ${\text{NH}}_{4}^{+}$, and negatively correlated with soil DOC and C:N ratios (Figure 2E). Fungal Shannon diversity was not significantly different between disturbed and reference forest soils (ANOVA *P* = 0.24, Figure 2D and Supplementary Figure S4B) but was positively correlated with soil pH (Figure 2E).

### Bacterial and Fungal β Diversity Patterns

**FIGURE 3:**
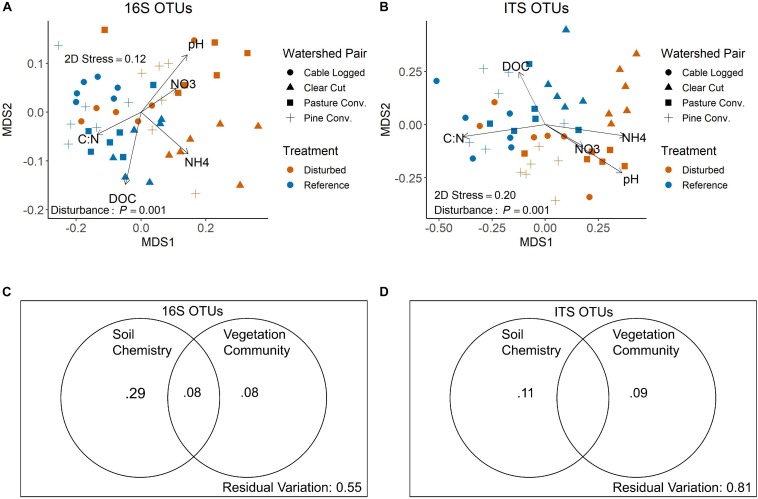
NMDS ordinations of 16S **(A)** and ITS **(B)** community structure at the OTU level. *P*-values shown are overall disturbance effects from PERMANOVA. Vectors display correlations of key soil variables with NMDS axes from envfit. Panels **C** and **D** display variation partitioning results for 16S **(C)** and ITS **(D)** communities. Values shown are adjusted *R*^2^-values for each respective partition, and all partitions shown are statistically significant (distance-based redundancy analysis *P* < 0.01). Adjusted *R*^2^-values < 0 not shown (i.e., middle partition on panel **D**).

NMDS visualization of bacterial communities using Bray–Curtis distances showed clear, statistically significant separation of communities based on past forest disturbance (Figure 3A, PERMANOVA *P* = 0.001). Additionally, all pairwise comparisons between disturbed-reference watershed pairs were significant (PERMANOVA, all adjusted *P* < 0.05). NMDS axis 1 was negatively correlated with soil C:N and DOC and positively correlated with soil ${\text{NO}}_{3}^{-}$, ${\text{NH}}_{4}^{+}$, and pH (Figure 3A). Variation partitioning showed soil chemistry accounting for 37% of observed variation in bacterial communities, 29% of which was independent of vegetation communities (Figure 3C). Vegetation communities accounted for 16% of observed variation in bacterial communities, 8% of which was independent of soil chemistry (Figure 3C). All partitions were statistically significant (distance-based redundancy analysis, all *P* < 0.01).

Similar to bacteria, NMDS visualization of fungal communities using Bray–Curtis distances showed statistically significant separation of communities based on past disturbance (Figure 3B, PERMANOVA *P* = 0.001). Pairwise comparisons were significant for the clear-cut, pine conversion, and pasture conversion watershed pairs (PERMANOVA, all adjusted *P* < 0.05), while the cable-logged watershed and its reference were marginally different (PERMANOVA adjusted *P* = 0.065). Similar to bacteria, fungal NMDS axis 1 was negatively correlated with soil C:N and DOC and positively correlated with soil ${\text{NO}}_{3}^{-}$, ${\text{NH}}_{4}^{+}$, and pH (Figure 3B). Variation partitioning for fungal communities showed soil chemistry accounting for 11% of observed fungal community variation and vegetation accounting for 9% of observed variation (Figure 3D) and all partitions were statistically significant (distance-based redundancy analysis, all *P* < 0.001).

### Bacterial Phyla, Fungal Classes, and Fungal Guilds

**FIGURE 4:**
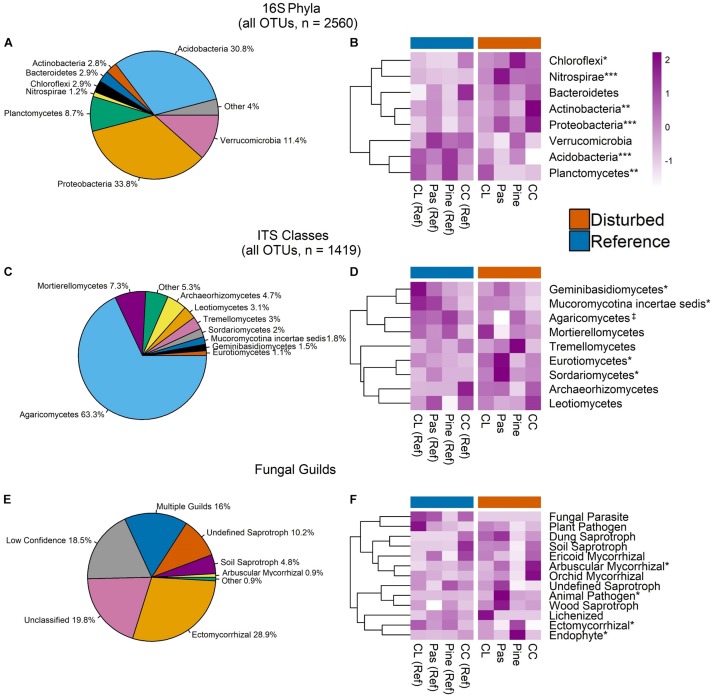
Pie charts display relative abundance of bacterial phyla **(A)**, fungal classes **(C)**, and fungal guilds **(E)** aggregated across all OTUs in all samples. Only phyla/classes comprising >1% of sequences and guilds comprising >0.9% of sequences are shown. Heat maps in panels **B**, **D**, and **F** show scaled *Z*-scores for relative abundances for each bacterial phylum **(C)**, fungal class **(D)**, and fungal guild **(F)** across each watershed. CC, clear-cut; CL, cable-logged; Pas, pasture conversion; Pine, pine conversion. Symbols represent statistical significance at the following levels: ^∗∗∗^*P* < 0.001, ^∗∗^*P* < 0.01, ^∗^*P* < 0.05, ^‡^*P* < 0.1. *P*-values are the main effects of disturbance from two-way ANOVA. Dendrograms on heat maps reflect similarity of relative abundance patterns of taxa across watersheds (complete-linkage clustering) and do not reflect phylogenetic relationships.

Aggregated across all samples, bacterial communities were dominated by the phyla Acidobacteria and Proteobacteria, which accounted for ∼65% of sequences (Figure 4A). Relative abundance of several bacterial phyla displayed disturbance effects, with Acidobacteria and Planctomycetes ∼20 and 12% higher in reference soils, respectively (ANOVA, both *P* < 0.01, Figure 4B). In contrast, Proteobacteria, Chloroflexi, Actinobacteria, and Nitrospirae had ∼11, 23, 39, and 280% higher relative abundance in disturbed soils, respectively (ANOVA, all *P* < 0.05, Figure 4B). Pairwise comparisons within disturbed-reference watershed pairs indicated that these patterns were consistent across all disturbances for Acidobacteria, Proteobacteria, and Nitrospirae (Supplementary Table S3). In contrast, disturbance effects for Planctomycetes and Chloroflexi were driven primarily by large differences in the pine conversion watershed pair, while effects for Actinobacteria were driven primarily by the clear-cut watershed pair (Supplementary Table S3).

Fungal communities were dominated by class Agaricomycetes, which had marginally higher relative abundance in reference forest soils (∼17% higher, ANOVA *P* = 0.07, Figure 4D) and comprised >63% of sequences (Figure 4C). Other fungal classes with significant disturbance effects include Geminibasidiomycetes and Mucoromycotina, which had 73 and 85% higher relative abundance in reference soils, respectively, while Sordariomycetes and Eurotiomycetes had 67 and 150% higher relative abundances in disturbed soils, respectively (ANOVA, all *P* < 0.05, Figure 4D). However, pairwise comparisons within disturbed-watershed pairs revealed that disturbance effects for each class were driven primarily by only one watershed pair, with differences in Agaricomycetes, Sordariomycetes, and Eurotiomycetes driven by large differences in the pasture conversion pair (Supplementary Table S4) and differences in Mucoromycotina and Geminibasidiomycetes driven by large differences in the cable-logged pair (Supplementary Table S4).

Analysis of fungal sequences using FUNGuild resulted in ∼46% of sequences confidently identified to a single functional guild (Figure 4E). Sequences identified as arbuscular mycorrhizae (AM) had ∼83% higher relative abundance in disturbed soils, while Ectomycorrhizae had ∼52% higher relative abundance in reference soils (ANOVA, both *P* < 0.05, Figure 4F). Pairwise comparisons within disturbed-reference watershed pairs showed that this pattern was consistent for both groups of mycorrhizae across all disturbances except for pine conversion, which showed the reverse patterns (i.e., higher Ectomycorrhizae and lower AM with pine conversion, Supplementary Table S5). Additionally, animal pathogens showed higher relative abundance with pasture conversion while endophytes showed higher relative abundance with pine conversion (ANOVA, both *P* < 0.05, Figure 4F and Supplementary Table S5).

### Differentially Abundant OTUs

**FIGURE 5:**
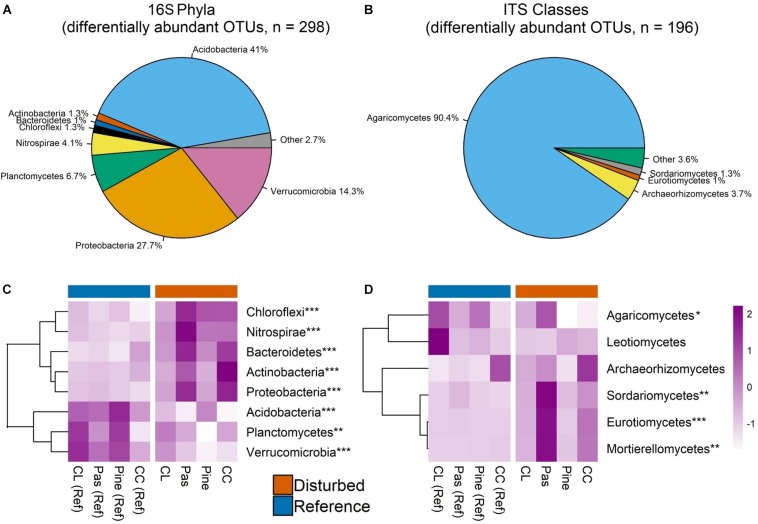
Pie charts display relative abundance of bacterial phyla **(A)** and fungal classes **(B)** aggregated across edgeR-identified differentially abundant OTUs. Only phyla/classes comprising >1% of sequences are shown. Heat maps in panels **C** and **D** show scaled *Z*-scores for relative abundances for each bacterial phylum **(C)** and fungal class **(D)** for differentially abundant OTUs across each watershed. CC, clear-cut; CL, cable-logged; Pas, pasture conversion; Pine, pine conversion. Asterisks represent statistical significance at the following levels: ^∗∗∗^*P* < 0.001, ^∗∗^*P* < 0.01, ^∗^*P* < 0.05. *P*-values are main effects of disturbance from two-way ANOVA. Dendrograms on heat maps reflect similarity of relative abundance patterns of taxa across watersheds (complete-linkage clustering) and do not reflect phylogenetic relationships.

EdgeR identified 298 bacterial OTUs as differentially abundant between disturbed and reference sites, ∼69% of which belonged to Acidobacteria and Proteobacteria (Figure 5A). Phylum-level analysis of these OTUs showed largely the same pattern as the full OTU dataset (Figures 4B, 5C), but disturbance effects for all phyla were significant (ANOVA, all *P* < 0.01, Figure 5C), indicating that differentially abundant bacterial taxa exhibit generally consistent disturbance responses at the phylum level. Additionally, though the pasture conversion and clear cut watershed pairs showed the largest pairwise differences, patterns of relative abundance were consistent across all watershed pairs for all phyla (Supplementary Table S6), indicating consistent phylum-level responses to different past disturbances.

EdgeR identified 196 fungal OTUs as differentially abundant between disturbed and reference sites, ∼90% of which belonged to class Agaricomycetes (Figure 5B). Class-level analysis of these OTUs showed that only Agaricomycetes, Sordariomycetes, Eurotiomycetes, and Motierellomycetes had significant disturbance effects (all *P* < 0.05, Figure 5D), and similar to the full OTU dataset, effects for each respective class were driven primarily by a single watershed pair (Supplementary Table S7), indicating that fungal disturbance responses were not consistent at the class level or across different past disturbances.

### Co-occurrence Networks

**FIGURE 6:**
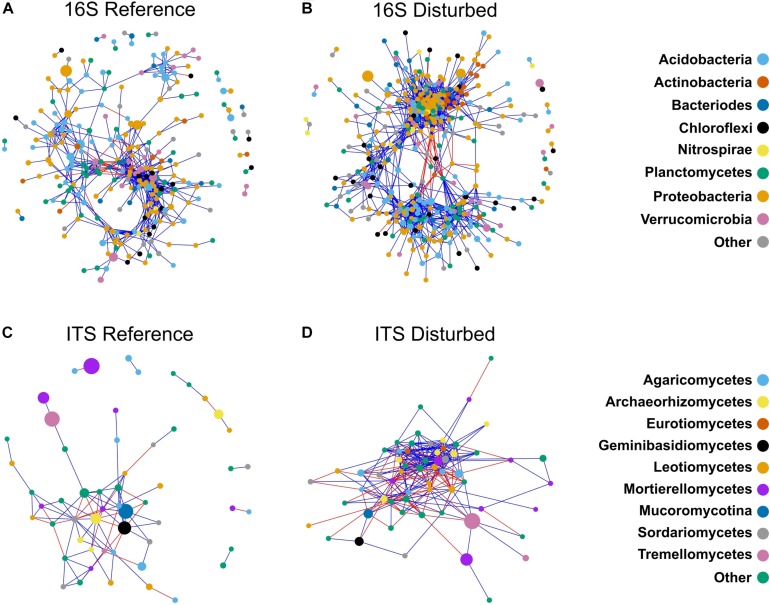
Co-occurrence networks constructed for bacterial communities in reference watersheds **(A)**, disturbed watersheds **(B)** and fungal communities in reference watersheds **(C)**, and disturbed watersheds **(D)**. Color of nodes is indicative of bacterial phylum **(A,B)** or fungal class **(C,D)**. Edge color is indicative of positive (blue), or negative (red) correlations. Size of nodes is proportional to relative abundance of the OTU represented.

Network analysis of bacterial communities showed distinct network topologies between reference and disturbed forest soils (Figures 6A,B). The disturbed network had more nodes (546 vs. 451) and edges (14,174 vs. 6,216) compared with reference communities (Figures 6A,B and Table 2), while the reference network had a higher proportion of negative edges than the disturbed network (0.15 vs. 0.09, *Z*-test *P* < 0.001, Table 2). The disturbed network had more connections per node (65% higher degree centrality), nodes that were closer on average to all other nodes in the network (10% higher closeness centrality), and nodes that were more tightly clustered together (20% higher clustering coefficient) than nodes in the reference network (all Kruskal–Wallis *P* < 0.001, Table 2). Also, the disturbed network had 28% lower betweenness centrality (Kruskal–Wallis *P* < 0.001, Table 2), indicating that nodes are less likely to bridge the shortest path between two nodes than nodes in the reference network.

**TABLE 2:** Network metrics calculated for 16S and ITS co-occurrence networks constructed for reference and disturbed soils.

	**Network metric**	**Reference**	**Disturbed**
Bacteria	Nodes	451	546
	Edges	6,216	14,174
	Prop. negative edges	0.148^∗∗∗^	0.087
	Degree centrality	0.047 (0.066)	0.077 (0.104)^∗∗∗^
	Betweenness centrality	0.003 (0.004)^∗∗∗^	0.002 (0.003)
	Closeness centrality	0.399 (0.067)	0.441 (0.07)^∗∗∗^
	Clustering coefficient	0.37 (0.14)	0.45 (0.18)^∗∗∗^
Fungi	Nodes	55	61
	Edges	95	279
	Prop. negative edges	0.284	0.333
	Degree centrality	0.037 (0.074)	0.15 (0.167)^∗∗∗^
	Betweenness centrality	0.002 (0.029)	0.013 (0.03)^∗^
	Closeness centrality	0.057 (0.039)	0.441 (0.127)^∗∗∗^
	Clustering coefficient	0.27 (0.5)	0.43 (0.23)^∗^

*For degree centrality, betweenness centrality, closeness centrality (all normalized), and clustering coefficient, values displayed are medians followed by interquartile ranges. Asterisks indicate significantly higher values at the following significance levels: ^∗^*P* < 0.05, ^∗∗∗^*P* < 0.001. Proportion of negative edges was compared between reference and disturbed soils using *Z*-tests, while all other metrics were compared using Kruskal–Wallis tests.*

Topologies were also distinct between reference and disturbed communities for fungal co-occurrence networks (Figures 6C,D). Similar to the bacterial networks, the disturbed fungal network had more nodes (61 vs. 55) and edges (279 vs. 95) than the reference network (Table 2). However, there was no difference in the proportion of negative edges between the disturbed and reference networks (*Z*-test, *P* = 0.45, Table 2) for fungi. Also similar to the bacterial networks, the disturbed fungal network had more connections per node (305% higher degree centrality), nodes that were closer to other nodes in the network (676% higher closeness centrality), and nodes that were more tightly clustered than in the reference network (all Kruskal–Wallis *P* < 0.05, Table 2). However, unlike the bacterial networks, the disturbed fungal network had 531% higher betweenness centrality (Kruskal–Wallis, *P* = 0.034, Table 2), indicating that nodes are more likely to bridge the shortest path between two nodes than nodes in the reference network.

## Discussion

### Bacterial Responses Are Consistent Across Different Disturbance Histories

Disturbance alters forest soil properties (e.g., increases ${\text{NO}}_{3}^{-}$ and pH, Supplementary Table S2) and processes (e.g., increases N-cycling rates) (Keiser et al., 2016), which we predicted would be associated with consistent long-term responses of soil bacterial communities across several different past disturbances. Our results are consistent with this prediction, as reference and disturbed bacterial communities were distinct in terms of community composition (Figure 3A), Shannon diversity (Figure 2B), copiotroph:oligotroph ratios (Figure 2C), and ITS:16S ratios (Figure 2A). Similar results were presented in a recent global meta-analysis of bacterial response to forest degradation (Zhou et al., 2018). Additionally, bacteria exhibited clear disturbance responses at high taxonomic levels (Figure 4B), and taxonomic analysis of differentially abundant OTUs revealed consistent patterns, with all dominant bacterial phyla (>1% of sequences) showing significant disturbance effects (Figure 5C and Supplementary Table S6). Importantly, the phylum Nitrospirae, a bacterial group involved in N-cycling processes (nitrite-oxidation, commamox), displayed particularly strong disturbance responses, with nearly threefold higher relative abundance in disturbed soils (Figure 4B). Bacterial co-occurrence networks also showed clear disturbance responses, with the disturbed network exhibiting more clustering, more connections among OTUs, and a lower proportion of negative correlations among OTUs (Figures 6A,B and Table 2) relative to the reference network. Thus, our network analyses suggest that disturbance affects not only the taxa present in soil bacterial communities but also alters potential ecological interactions among bacterial taxa. For example, reductions in negative correlations in the disturbed network may reflect fewer competitive interactions between bacterial taxa due to relaxation of nutrient limitation with increased inorganic-N availability. Overall, our results suggest that disturbance of forests in the Appalachian region fundamentally alters bacterial community structure and ecological interactions over decadal time scales and that these changes are consistent across a range of disturbance types, including agricultural conversion, conversion to timber plantation, and commercial clear-cutting.

The observed bacterial community metrics were strongly correlated with several soil properties (e.g., pH, inorganic-N, C:N ratios, Figure 2E), with soil chemistry accounting for 37% of observed variation in bacterial community structure (Figure 3C), consistent with previous studies demonstrating that soil physicochemical properties are the primary drivers of soil bacterial communities (e.g., Fierer and Jackson, 2006; Lauber et al., 2008, 2009). The differences in soil properties observed in this study are likely linked to vegetation changes that occur during forest succession following disturbance. Early successional forests in this region are often dominated by N-fixing black locust (*Robinia pseudoacacia*) (Elliott and Vose, 2011), which likely contributed to increased inorganic-N levels and lower soil C:N in disturbed watersheds. Vegetation differences may also be responsible for soil pH shifts, as previously disturbed watersheds in this study were associated with reduced abundance of species with acidic leaf litter such as rosebay rhododendron (*Rhododendron maximum*) and oaks (*Quercus* spp.) and increased abundance of species with higher pH litter such as red maple (*Acer rubrum*) and tulip poplar (*Liriodendron tulipifera*) (Supplementary Figure S1), which over long time scales may have contributed to increased soil pH. In addition to vegetation changes, the pasture conversion watershed was limed in 1959, likely explaining the relatively high soil pH (∼5.75) and clear disturbance effects on bacterial communities we observed for this site, including the highest observed bacterial Shannon diversity of all examined watersheds (Figure 2B).

Although bacterial responses to disturbance were largely consistent, we also observed some responses that varied among watersheds with different past disturbances. For example, changes in Chloroflexi and Planctomycetes were only observed in the pine conversion pair, while changes in Actinobacteria were only observed in the clear-cut pair (Figure 4B). These context-dependent effects may be related to unique effects of specific disturbances on plant communities observed in this study (e.g., conversion to pine monoculture, Supplementary Figure S1), as vegetation accounted for a significant proportion of variation in bacterial communities, 8% of which was independent of soil chemistry (Figure 3C). Although some prior studies have not found strong correlations between vegetation and soil bacterial communities (e.g., Fierer and Jackson, 2006; Jangid et al., 2011), our results suggest that these relationships may indeed exist and therefore vegetation should be considered when assessing responses of soil bacteria to environmental change.

### Fungal Responses Vary Among Different Disturbance Histories

Fungal communities also showed evidence of long-term responses to past forest disturbance, with distinct fungal community composition between reference and disturbed soils (Figure 3B). Similar to bacteria, disturbed and reference fungal communities displayed distinct co-occurrence patterns, with the disturbed network displaying higher clustering and more connections among fungal OTUs (Figures 6C,D and Table 2). However, other fungal community metrics did not have consistent disturbance responses; fungal Shannon diversity was not different between reference and disturbed soils and disturbance responses were not consistent at the class level for the full OTU dataset (Figure 4C and Supplementary Table S4) or for differentially abundant OTUs (Figure 5D and Supplementary Table S7). The inconsistent responses we observed for fungi at the class level likely reflect the diversity of life strategies that occur within fungal classes. Another potential explanation for the observed responses is that fungal communities have high fidelity to plant communities (Bonfante and Anca, 2009; Peay et al., 2013), and watersheds with different past disturbances sampled for this study displayed unique plant communities (Supplementary Figure S1). Indeed, vegetation communities accounted for a significant proportion (9%) of observed variation in fungal communities (Figure 3D) and other studies in temperate forests have also noted the importance of vegetation in structuring fungal communities (e.g., Goldmann et al., 2015). Analysis of fungal functional guilds also reflects the importance of vegetation in structuring fungal communities; our disturbed sites had lower abundance of tree species that host ECM fungi such as oaks (i.e., *Quercus montana*, Supplementary Figure S1), and our disturbed fungal communities indeed displayed lower relative abundance of ECM fungi (Figure 4F), with the exception of conversion to white pine (*Pinus strobus*) (Figure 4F), which is a known ECM host. Additionally, tree species that host AM, such as red maple (*A. rubrum*) and tulip poplar (*L. tulipifera*), were more abundant in our previously disturbed sites, and these communities featured higher relative abundance of AM fungi (Figure 4F), with the exception of conversion to pine (Figure 4F).

Soil properties also likely played a role in structuring fungal communities; soil chemistry accounted for a significant proportion of variation (11%) in fungal community composition (Figure 3D), and previous studies have found nutrient status (e.g., soil C:N) to be an important driver of soil fungal communities (Lauber et al., 2008). Although it is likely that both soil physicochemical properties and vegetation are important in structuring fungal communities, we were able to explain much less variation in fungal communities (∼20% of variation explained, Figure 3C) relative to bacterial communities (∼45% of variation explained, Figure 3D), suggesting that factors we did not consider, such as soil phosphorus, herbaceous vegetation, and elevation may be important in determining fungal community structure, as has been shown in other studies from temperate forests in the Appalachian region (Veach et al., 2017). Additionally, variation partitioning analysis of fungal guilds increased the proportion of explained variation in fungal communities (∼40% of variation explained, Supplementary Figure S5), highlighting the potential usefulness of trait-based approaches for describing fungal communities in addition to taxonomy-based approaches.

### Community Shifts Contribute to Altered N-Cycling After Disturbance

In addition to documenting community shifts, our results cast new light on the role of soil microbial communities in long-term biogeochemical responses to disturbances that have been observed in Appalachian forests (e.g., Webster et al., 2016). Existing frameworks such as the mycorrhizal-associated nutrient economy (MANE) (Phillips et al., 2013) have been used to predict temperate forest N-cycling rates using known tree-mycorrhizal associations (ECM vs. AM), informed by differences in nutrient acquisition pathways of ECM vs. AM fungi. This framework predicts that forests dominated by trees with AM symbionts (e.g., maple, tulip poplar) will feature rapid N-cycling rates and soil N pools dominated by inorganic-N. These predictions are generally consistent with our results for mycorrhizal fungi (Figure 4F) and vegetation communities (Supplementary Figure S1), and with previous studies on soil N-cycling (e.g., Keiser et al., 2016) from the region. However, our results suggest that the MANE framework and associated trees/mycorrhizae are part of a complex system of feedbacks in Appalachian forests that also includes land use history, forest successional dynamics, and soil bacterial communities. In these ecosystems, N-fixing black locust often dominates plant communities following disturbance (Elliott and Vose, 2011), increasing soil inorganic-N and likely promoting increased abundance of copiotrophic bacterial taxa (Ramirez et al., 2012; Figure 2B). Some bacterial copiotrophs have been linked to elevated N-mineralization rates in soil (Fierer et al., 2007), which may contribute to persistently elevated inorganic-N pools even after successional declines of N-fixers. Higher inorganic-N facilitates N-acquisition by AM fungi, potentially promoting dominance of their maple and poplar hosts in disturbed sites, and the high pH litter of these tree species likely leads to higher pH in disturbed soils over long time scales. Increased soil pH further alters soil bacterial communities, including increased abundance of bacterial nitrifiers such as ammonia-oxidizers (Stempfhuber et al., 2015; Lin et al., 2017) and nitrite oxidizers (i.e., Nitrospirae, Figure 4B), likely resulting in elevated nitrification rates in soil (Norman and Barrett, 2014; Keiser et al., 2016) and persistently increased rates of nitrate export from these previously disturbed watersheds (Swank and Vose, 1997; Webster et al., 2016). The long-term responses we observed may not be universal across temperate forests, as other studies have reported different long-term forest N-cycle disturbance responses; for example, a previously disturbed forest in the northern Appalachians displayed reduced N-cycling rates relative to a reference forest (Goodale and Aber, 2001). However, similar N-cycle responses to forest disturbance have been documented in several forests across the continental United States (Vitousek et al., 1979), suggesting that similar microbial community responses may be expected across temperate forest ecosystems, at least in the short term.

## Conclusion

Overall, our results show different long-term responses of bacterial and fungal communities to forest disturbance in Appalachian forests of the Eastern United States. A similar study from the region also showed distinct responses of bacteria and fungi along a forest recovery chronosequence following mine reclamation (Sun et al., 2017), further suggesting that different microbial groups will respond differently to environmental change. Additionally, we noted striking differences in co-occurrence network characteristics between both bacterial and fungal communities in reference and disturbed soils. For both groups, disturbed communities showed more connected, clustered, and overall more complex networks (Figure 6 and Table 2). Although co-occurrence patterns do not necessarily imply ecological relationships (Faust and Raes, 2012), our networks suggest the possibility of fundamentally altered microbial community interactions following disturbance. For example, the more complex networks observed in disturbed soils suggest these soils are potentially characterized by more microbial interactions and overall higher biological activity (Karimi et al., 2017), which is supported by direct assays of microbial activity from these sites (i.e., higher SIR for disturbed soils, Supplementary Table S2). Additionally, our networks show more potential negative interactions for fungi vs. bacteria and for reference bacteria vs. disturbed bacteria. Previous studies have suggested that negative ecological interactions (i.e., competition) increase microbial community stability under environmental change (Coyte et al., 2015), suggesting that fungal communities will be more resilient to perturbations (i.e., drought, warming) than bacterial communities, similar to observations in a grassland ecosystem (De Vries et al., 2018), and that reference bacterial communities will be more resilient than disturbed bacterial communities. Evaluating these hypotheses should be a priority for future research, as temperate forests are already experiencing stresses associated with climate change (i.e., increased drought frequency and severity) (Burt et al., 2018), likely altering the structure and biogeochemical functions of soil microbial communities and potentially threatening the critical ecosystem services they provide for the region.

## Data Availability Statement

The datasets generated for this study can be found in the NCBI BioProject Database under accession number PRJNA548911 and the author’s GitHub repository found at https://github.com/eosburn/Coweeta-Microbes.

## Author Contributions

EO, BB, BS, FA, JK, and JB contributed to the study design. EO conducted the field sampling and sample preparation/analysis. EO, SM, and FA contributed to the statistical analyses. All authors contributed to the writing and editing of the manuscript.

## Conflict of Interest

The authors declare that the research was conducted in the absence of any commercial or financial relationships that could be construed as a potential conflict of interest.
