# Supplementary material for: Soil Bacterial and Fungal Communities Exhibit Distinct Long-Term Responses to Disturbance in Temperate Forests
Source: Front Microbiol. 2019 Dec 11;10:2872. doi: 10.3389/fmicb.2019.02872 (PMC6917579; doi:10.3389/fmicb.2019.02872)
Supplement: Supplementary file 1 [file Table_1.DOCX]

**Soil bacterial and fungal communities exhibit distinct long-term responses to forest disturbance in temperate ecosystems**

Supplementary Material

**Table S1:** Detailed watershed information for all eight watersheds sampled in this study. Note that dominant vegetation (by density) is for study plots only and is not intended to represent watershed-scale woody vegetation communities.

| **Watershed Pair** | **Watershed Number** | **Treatment** | **Aspect** | **Elevation (m)** | **Area (ha)** | **Dominant Woody Vegetation** |
| --- | --- | --- | --- | --- | --- | --- |
| Cable Logged | WS2 | Undisturbed since 1923 | SSE | 709 – 1004 | 12 | *Rhododendron maximum, Quercus spp., Betula spp.* |
|  | WS7 | Commercially clear cut and cable logged in 1977 | S | 772 – 1077 | 59 | *Rhododendron maximum, Quercus montana., Acer rubrum, Liriodendron tulipifera* |
| Pasture Conversion | WS14 | Undisturbed since 1923 | NW | 707 – 992 | 61 | *Rhododendron maximum, Liriodendron tulipifera, Betula spp.* |
|  | WS6 | Clearcut in 1958, soil scarified, planted to grass, limed and fertilized in 1959, fertilized again in 1965, grass herbicided in 1966 and 1967 | NW | 696 – 905 | 9 | *Acer rubrum, Liriodendron tulipifera* |
| Pine Conversion | WS18 | Undisturbed since 1923 | NW | 726 – 993 | 13 | *Rhododendron maximum, Acer rubrum, Betula spp.* |
|  | WS17 | All woody vegetation cut annually 1940 - 1955, white pine planted in 1956 | NW | 760 – 1021 | 13 | *Pinus strobus* |
| Clear Cut | WS36 | Undisturbed since 1923 | ESE | 1021 – 1542 | 49 | *Rhododendron maximum, Liriodendron tulipifera, Betula spp.* |
|  | WS37 | All woody vegetation cut in 1963, no products removed | ENE | 1033 – 1592 | 44 | *Liriodendron tulipifera, Betula spp.* |

Additional watershed information can be found at: <https://coweeta.uga.edu/sitehistory/basin>

**Table S2:** Soil physicochemical variables aggregated across all disturbed and reference soil samples. Means ± one SE shown. Asterisks indicate significantly higher values (ANOVA) at the following significance levels: * *P* < 0.05, ** *P* < 0.01, *** *P* < 0.001.

| **Variable** | **Reference** | **Disturbed** |
| --- | --- | --- |
| pH | 5.25 (0.038) | 5.56 (0.052)*** |
| Moisture (g H_2_O g soil^-1^) | 0.30 (0.014) | 0.294 (0.008) |
| NO_3_ (µg N g soil^-1^) | 0.119 (0.040) | 1.08 (0.443)*** |
| NH_4_ (µg N g soil^-1^) | 1.79 (0.140) | 2.53 (0.187)*** |
| DOC (µg C g soil^-1^) | 391.9 (13.0)*** | 302.7 (17.7) |
| TDN (µg N g soil^-1^) | 46.7 (2.41) | 42.8 (2.59) |
| DOC:TDN  DON (µg N g soil^-1^) | 8.61 (0.264)*  44.7 (2.30)* | 7.22 (0.286)  39.1 (2.50) |
| Microbial Biomass C (µg C g soil^-1^) | 207.3 (13.3)* | 176.0 (15.8) |
| Microbial Biomass N (µg N g soil^-1^)  Microbial Biomass C:N  Total C (mg C g soil^-1^) | 41.5 (2.62)  4.85 (0.174)*  41.3 (3.18) | 40.0 (3.27)  4.30 (0.115)  45.5 (4.95) |
| Total N (mg N g soil^-1^) | 2.35 (0.203) | 2.90 (0.328)* |
| C:N | 17.9 (0.440)** | 16.1 (0.354) |
| SIR (µg CO_2_-C g soil^-1^ d^-1^) | 82.8 (4.13) | 93.1 (7.52)* |

**Table S3:** Pairwise comparisons between watersheds within disturbed-reference pairs for bacterial phyla (all OTUs) showing main effects of disturbance on relative abundance. Means ± one SE shown. Asterisks indicate significantly higher values at the following significance levels: * *P* < 0.05, ** *P* < 0.01, *** *P* < 0.001.

| Bacterial Phylum | Cable Logged | | Pasture Conversion | | Pine Conversion | | Clear Cut | |
| --- | --- | --- | --- | --- | --- | --- | --- | --- |
|  | Reference | Disturbed | Reference | Disturbed | Reference | Disturbed | Reference | Disturbed |
| Acidobacteria | 0.345 (0.012) | 0.310 (0.009) | 0.319** (0.013) | 0.267 (0.014) | 0.366** (0.012) | 0.311 (0.009) | 0.309*** (0.016) | 0.233 (0.016) |
| Proteobacteria | 0.310 (0.008) | 0.332 (0.005) | 0.337 (0.006) | 0.367* (0.014) | 0.303 (0.012) | 0.336* (0.015) | 0.331 (0.010) | 0.389*** (0.006) |
| Nitrospirae | 0.007 (0.001) | 0.013 (0.005) | 0.005 (0.002) | 0.030*** (0.005) | 0.004 (0.002) | 0.017** (0.002) | 0.005 (0.001) | 0.017* (0.004) |
| Planctomycetes | 0.097 (0.004) | 0.097  (0.005) | 0.085 (0.005) | 0.077 (0.006) | 0.102**  (0.011) | 0.076 (0.004) | 0.083 (0.003) | 0.079 (0.007) |
| Chloroflexi | 0.026 (0.005) | 0.029 (0.004) | 0.024 (0.004) | 0.03 (0.003) | 0.024 (0.003) | 0.038* (0.004) | 0.031 (0.002) | 0.031 (0.002) |
| Actinobacteria | 0.023 (0.002) | 0.024 (0.002) | 0.028 (0.003) | 0.030 (0.004) | 0.016 (0.003) | 0.019 (0.002) | 0.026 (0.005) | 0.056*** (0.006) |

**Table S4:** Pairwise comparisons between watersheds within disturbed-reference pairs for fungal classes (all OTUs) showing main effects of disturbance on relative abundance. Means ± one SE shown. Asterisks indicate significantly higher values at the following significance levels: * *P* < 0.05, ** *P* < 0.01, *** *P* < 0.001.

| Fungal Class | Cable Logged | | Pasture Conversion | | Pine Conversion | | Clear Cut | |
| --- | --- | --- | --- | --- | --- | --- | --- | --- |
|  | Reference | Disturbed | Reference | Disturbed | Reference | Disturbed | Reference | Disturbed |
| Agaricomycetes | 0.676 (0.057) | 0.619 (0.055) | 0.665 (0.044) | 0.516 (0.043) | 0.711 (0.035) | 0.666 (0.022) | 0.618 (0.043) | 0.595 (0.096) |
| Sordariomycetes | 0.013 (0.003) | 0.021 (0.006) | 0.021 (0.006) | 0.039* (0.004) | 0.014 (0.003) | 0.018 (0.005) | 0.014 (0.002) | 0.020 (0.006) |
| Eurotiomycetes | 0.011 (0.004) | 0.016 (0.004) | 0.007 (0.002) | 0.032*** (0.011) | 0.004 (0.0004) | 0.002 (0.001) | 0.022 (0.0004) | 0.011 (0.005) |
| Mucoromycotina | 0.040* (0.009) | 0.019 (0.004) | 0.034 (0.010) | 0.018 (0.009) | 0.016 (0.003) | 0.012 (0.002) | 0.004 (0.001) | 0.001 (0.0004) |
| Geminibasidiomycetes | 0.040*** (0.011) | 0.010 (0.004) | 0.020 (0.004) | 0.022 (0.003) | 0.009 (0.001) | 0.010 (0.002) | 0.004 (0.001) | 0.002 (0.001) |

**Table S5:** Pairwise comparisons between watersheds within disturbed-reference pairs for fungal guilds showing main effects of disturbance on relative abundance. Means ± one SE shown. Asterisks indicate significantly higher values at the following significance levels: * *P* < 0.05, ** *P* < 0.01, *** *P* < 0.001.

| Fungal Guild | Cable Logged | | Pasture Conversion | | Pine Conversion | | Clear Cut | |
| --- | --- | --- | --- | --- | --- | --- | --- | --- |
|  | Reference | Disturbed | Reference | Disturbed | Reference | Disturbed | Reference | Disturbed |
| Ectomycorrhizal | 0.475* (0.082) | 0.292 (0.062) | 0.298** (0.066) | 0.067 (0.030) | 0.412 (0.084) | 0.538 (0.039) | 0.195 (0.069) | 0.022 (0.019) |
| Arbuscular Myco. | 0.003 (0.002) | 0.006 (0.001) | 0.006 (0.002) | 0.013 (0.003) | 0.006 (0.002) | 0.003 (0.001) | 0.010 (0.001) | 0.021** (0.005) |
| Animal Pathogen | 0 | 0.0003 (0.0003) | 0 | 0.005*** (0.002) | 0.0002 (0.0001) | 0.001 (0.001) | 0 | 0.005 (0.002) |
| Endophyte | 0.001 (0.0005) | 0.004 (0.002) | 0.001 (0.001) | 0.001 (0.001) | 0.001 (0.0005) | 0.016*** (0.005) | 0.004 (0.002) | 0.001 (0.001) |

**Table S6:** Pairwise comparisons between watersheds within disturbed-reference pairs for bacterial phyla (differentially abundant OTUs) showing main effects of disturbance on relative abundance. Means ± one SE shown. Asterisks indicate significantly higher values at the following significance levels: * *P* < 0.05, ** *P* < 0.01, *** *P* < 0.001.

| Bacterial Phylum | Cable Logged | | Pasture Conversion | | Pine Conversion | | Clear Cut | |
| --- | --- | --- | --- | --- | --- | --- | --- | --- |
|  | Reference | Disturbed | Reference | Disturbed | Reference | Disturbed | Reference | Disturbed |
| Acidobacteria | 0.434 (0.006) | 0.405 (0.023) | 0.456*** (0.015) | 0.315 (0.028) | 0.466 (0.023) | 0.442 (0.009) | 0.479*** (0.015) | 0.327 (0.024) |
| Proteobacteria | 0.226 (0.009) | 0.273* (0.009) | 0.250 (0.013) | 0.333*** (0.019) | 0.221 (0.036) | 0.275* (0.006) | 0.247 (0.010) | 0.358*** (0.017) |
| Nitrospirae | 0.023 (0.005) | 0.040 (0.015) | 0.017 (0.007) | 0.089*** (0.011) | 0.012 (0.005) | 0.057*** (0.007) | 0.019 (0.004) | 0.049* (0.008) |
| Planctomycetes | 0.089 (0.004) | 0.076 (0.006) | 0.071 (0.008) | 0.055 (0.007) | 0.088*** (0.012) | 0.044 (0.007) | 0.056 (0.007) | 0.059 (0.004) |
| Chloroflexi | 0.010 (0.003) | 0.012 (0.003) | 0.008 (0.002) | 0.019** (0.004) | 0.009 (0.002) | 0.019** (0.002) | 0.006 (0.002) | 0.017** (0.002) |
| Actinobacteria | 0.001 (0.001) | 0.007 (0.003) | 0.002 (0.001) | 0.022*** (0.006) | 0.001 (0.0003) | 0.007 (0.002) | 0.008 (0.002) | 0.044*** (0.005) |
| Verrucomicrobia | 0.198* (0.010) | 0.153 (0.017) | 0.175*** (0.014) | 0.104 (0.011) | 0.187*** (0.022) | 0.097 (0.003) | 0.155*** (0.008) | 0.097 (0.010) |
| Bacteroidetes | 0.001 (0.001) | 0.010 (0.004) | 0.002 (0.001) | 0.021*** (0.004) | 0 | 0.012* (0.003) | 0.011 (0.006) | 0.020* (0.004) |

**Table S7:** Pairwise comparisons between watersheds within disturbed-reference pairs for fungal classes (differentially abundant OTUs) showing main effects of disturbance on relative abundance. Means ± one SE shown. Asterisks indicate significantly higher values at the following significance levels: * *P* < 0.05, ** *P* < 0.01, *** *P* < 0.001.

| Fungal Class | Cable Logged | | Pasture Conversion | | Pine Conversion | | Clear Cut | |
| --- | --- | --- | --- | --- | --- | --- | --- | --- |
|  | Reference | Disturbed | Reference | Disturbed | Reference | Disturbed | Reference | Disturbed |
| Agaricomycetes | 0.943 (0.031) | 0.868 (0.072) | 0.956 (0.018) | 0.873 (0.059) | 0.864 (0.088) | 0.833 (0.079) | 0.873* (0.059) | 0.634 (0.105) |
| Sordariomycetes | 0.001 (0.0003) | 0.010 (0.008) | 0.014 (0.007) | 0.036 (0.009) | 0.005 (0.004) | 0.012 (0.006) | 0.001 (0.001) | 0.035** (0.016) |
| Eurotiomycetes | 0.0001 (0.0001) | 0.006 (0.003) | 0.0004 (0.0003) | 0.036** (0.019) | 0 | 0.002 (0.002) | 0 | 0.028* (0.011) |
| Mortierellomycetes | 0 | 0.002 (0.001) | 0 | 0.011** (0.005) | 0.0003 (0.0003) | 0.001 (0.001) | 0.001 (0.001) | 0.007 (0.004) |


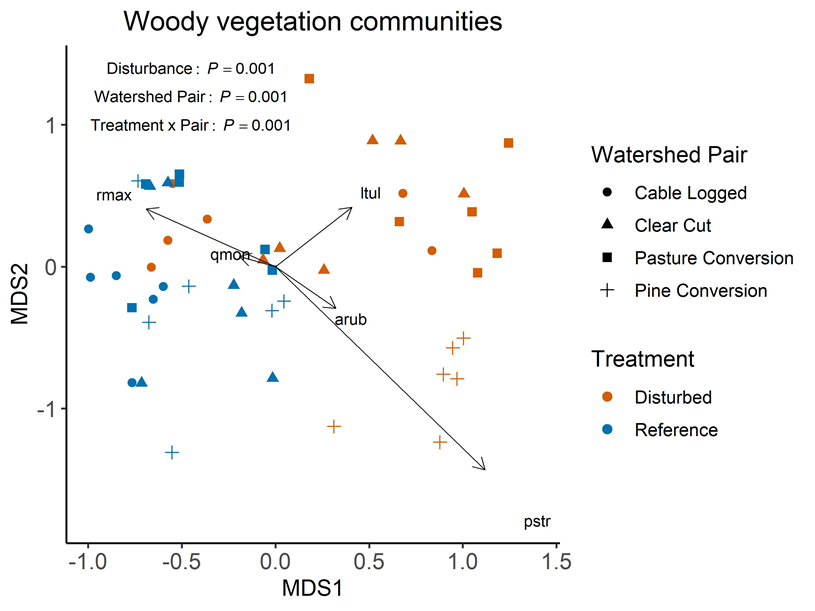


**Figure S1:** NMDS ordination of woody vegetation community structure. P-values shown are from PERMANOVA. Vectors shown are species scores for key taxa. Abbreviations are as follows: *Rhododendron maximum* (rmax), *Quercus montana* (qmon), *Liriodendron tulipifera* (ltul), *Acer rubrum* (arub), *Pinus strobus* (pstr).

**
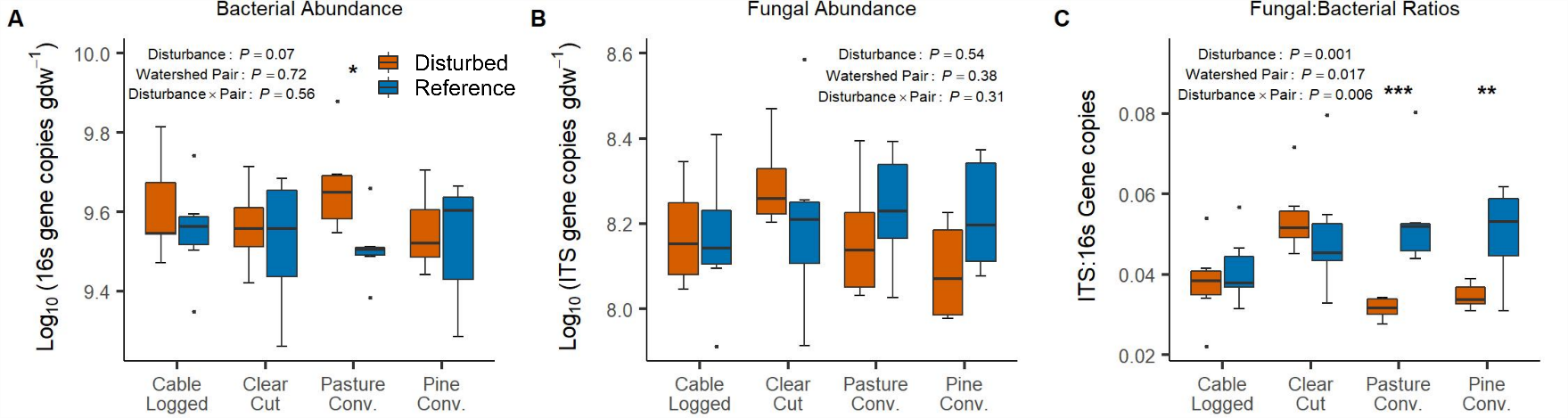
**

**Figure S2:** Pairwise comparisons of watersheds within each disturbed-reference pair for 16S gene copy abundance (A), ITS gene copy abundance (B), and ITS:16S gene copy ratios (C). Asterisks represent statistical significance at the following levels: *** *P* < 0.001, ** *P* < 0.01, * *P* < 0.05.


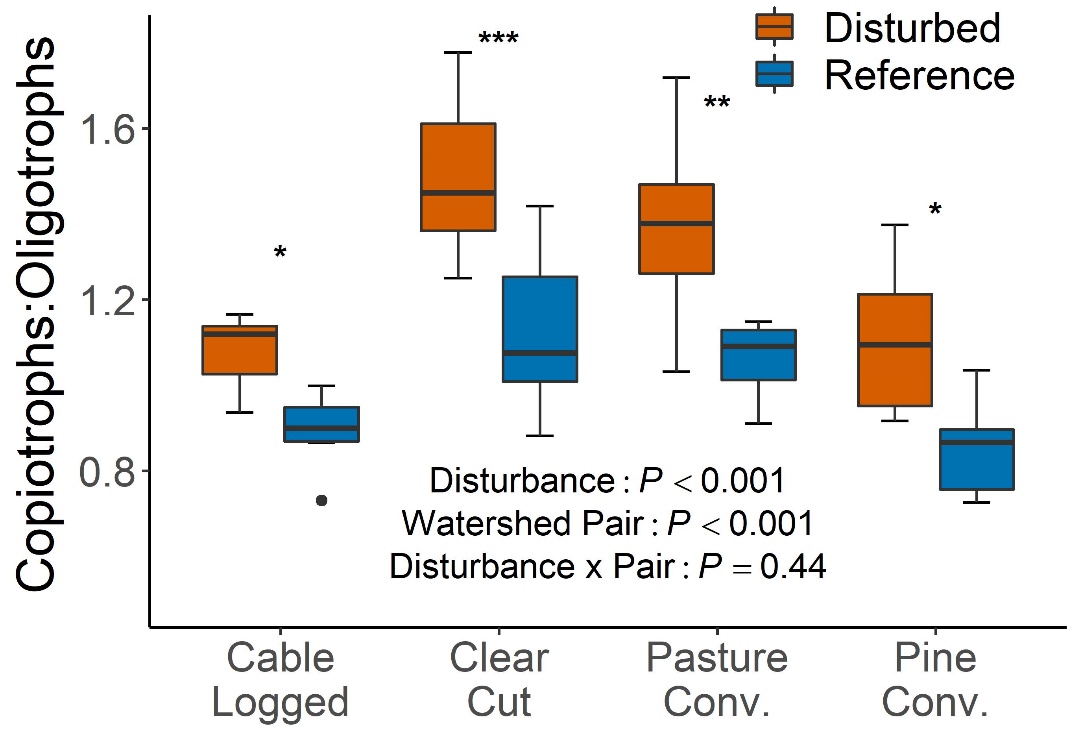


**Figure S3:** Pairwise comparisons of watersheds within each disturbed-reference pair Copiotroph:Oligotroph ratios. Asterisks represent statistical significance at the following levels: *** *P* < 0.001, ** *P* < 0.01, * *P* < 0.05


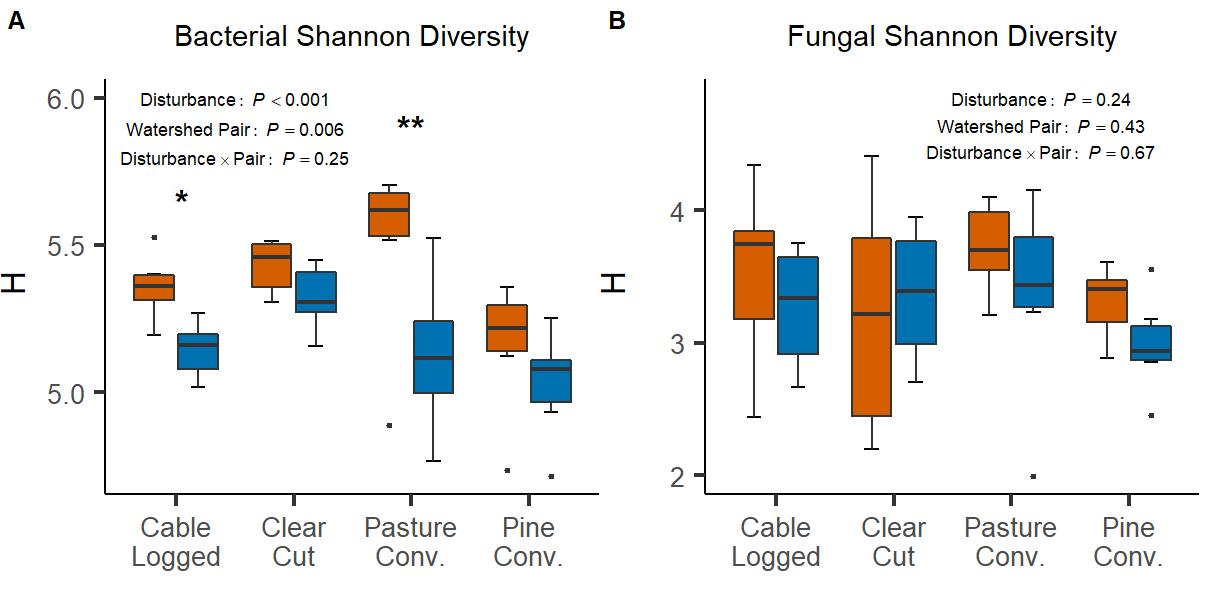


**Figure S4:** Pairwise comparisons of watersheds within each disturbed-reference pair for 16S OTU Shannon diversity (A), ITS OTU Shannon diversity (B). Asterisks represent statistical significance at the following levels: *** *P* < 0.001, ** *P* < 0.01, * *P* < 0.05.


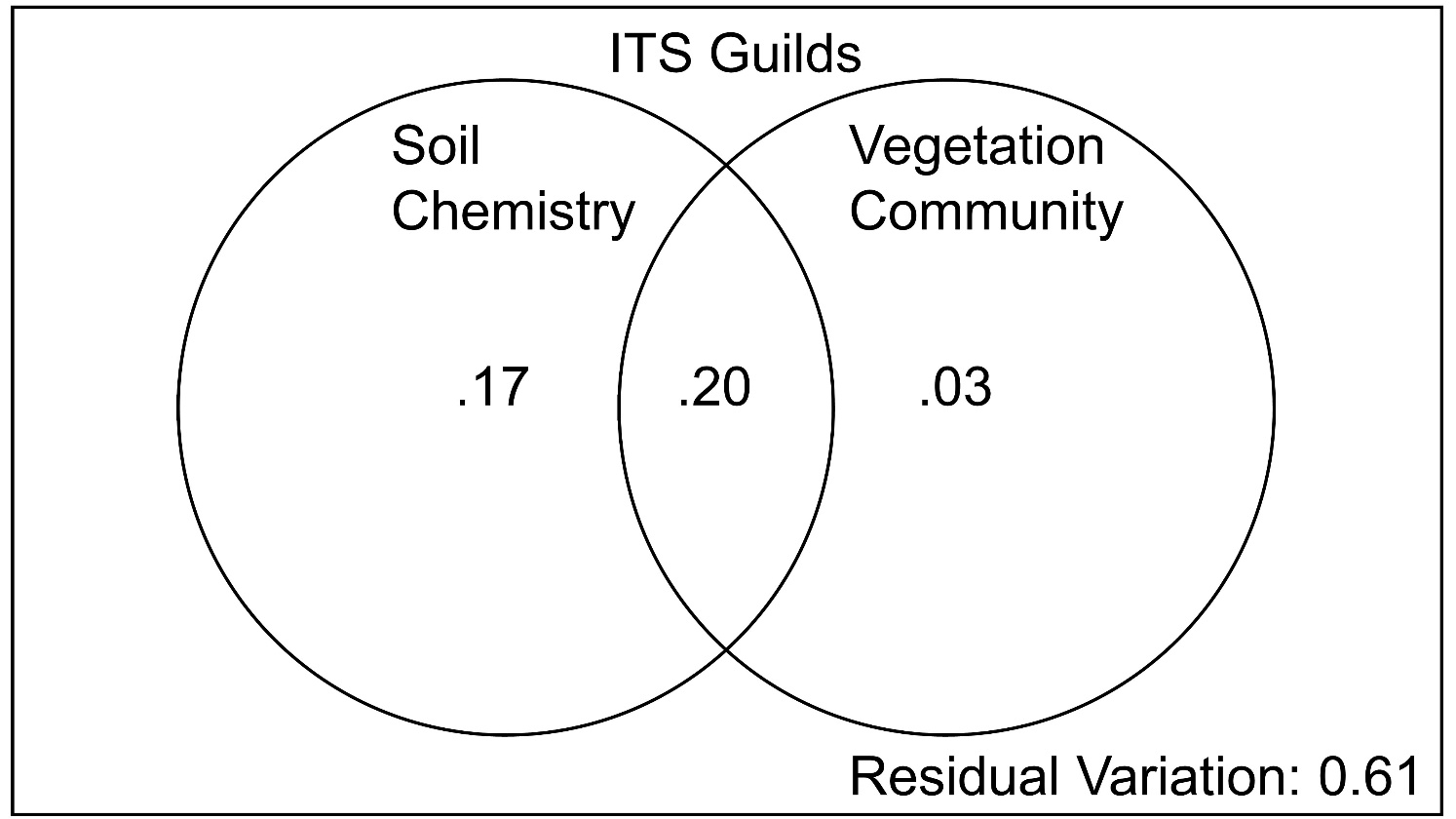


**Figure S5:** Variation partitioning for fungal guilds. Asterisks represent statistical significance at the following levels: *** *P* < 0.001, ** *P* < 0.01, * *P* < 0.05. Values shown are adjusted R^2^ values for each respective partition, and all partitions shown are statistically significant (distance-based redundancy analysis *P* < 0.01). Adjusted R^2^ values < 0 not shown.
